# The Library of the Chirurgeon

**Published:** 1894-06-30

**Authors:** 


### The First Century of English Medical Literature.

VI.—THE LIBRARY OF THE CHIRURGEON.

Among the best esteemed works on surgery in the sixteenth century was the treatise known as the "Questyonary of Cyrurgyers," translated from the French of Guido de Chauliac, first by Robert Copland (1542), and again by George Baker in 1579 under the title of "Guydo's Questions." Guido de Chauliac, a physician of Montpellier, and later on attached to the service of three successive Popes, flourished about the middle of the fourteenth century. He earned the title of the Restorer of Surgery, not only by reviving the precepts of the Greek and Arabian surgeons, long fallen into oblivion, but by many important discoveries, the result of a lifetime spent in observation and practice. His great work on surgery was for a long time the surgeons' only text-book. His Questyonary comprises a complete anatomy so far as the human frame was then understood, in the form of question and answer; it was probably intended to be learnt by heart, but it must be extremely doubtful whether the young student ever gained from it any very clear ideas. A short extract from Copland's early translation will serve to show the standard of physiological science at that time. "How many ears hath the heart, and how are they set, and whereof serve they? The heart hath two ears, on each side one, set upon the said lateral ventricles; they serve for to let the air in and out that is apparelled for it from the lungs. Whereof serveth a cartilaginous bone that is in the heart? It is to stay and strength it. Whereof is the substaunce of the heart? It is called precordium; it is of a skinny substaunce whereto descendeth sinews as unto other inward entrails. With what members hath the heart collygance? With all members and especially with the lungs wherewith it is bound. And with the mediastinum wherewith it is stayed and strengthened. May the heart sustain disease long? No, by reason of his great dignity."

For more than a century after the death of Guido surgery may be said to have made no perceptible advance. But the sixteenth century saw the rise of many eminent men, who lent to surgery the learning they had acquired as physicians, and assisted in raising it abroad (though not yet in England) above the contempt attaching to a mere mechanical *tour de force*. Their works were eagerly translated into English, and had no inconsiderable effect in causing English surgeons to recognise their ignorance, and open their eyes.

Antoine Chaumette, a learned physician and surgeon of Montpellier, was translated by Banister in 1575, with a set of dedicatory verses to "learned dames," from which it may be inferred that surgery was thought an appropriate accomplishment for ladies, even by surgeons themselves.

Tagalt, a Parisian doctor, who died in 1545, is chiefly noted as one of the first who paid surgery the doubtful compliment of writing on it in Latin. He also was translated by Banister. The Florentine Morus, whom Tagalt imitated, was translated by Richard Caldwell, President of the Royal College of Physicians, and founder of a surgery lecture in the College, which, however, few or none of the students would consent to attend.

Franciscus Arcæus, who practised medicine and surgery in Spain, and wrote a treatise on surgery at the age of eighty in 1573, was translated by Read. He was in advance of his times in urging the abandonment of plugging in simple wounds, and was celebrated for his success in trepanning. He gives interesting examples of his practice, illustrating the special dangers of the times, when, if complicated injuries from machinery had not yet begun to baffle the surgeon, he was liable at any minute to be called to a patient "wounded with a broad dagger four times behind his back," or "in his breast with somewhat a long sword which they call Verdugun."

A sensible little treatise on surgery was translated by S. Hobbes, in 1596, from the Latin of Cornelius Schilander, containing "A brief method of curing wounds and ulcers." The author frankly explains that he reserves his best prescriptions, "which are not rashly to be published to all," and being rallied on this account, admits that he will not communicate his secrets to others "because he would be rich himself." The book is, perhaps, on this very account, singularly free from absurdities and quackery.

More popular than these sober writers was Leonard Fioravanti, who practised medicine and surgery at Bologna, and was something of an empiric in his methods, though he has the credit of reprehending the common abuse of bleeding. His treatise on surgery was translated by John Hester in 1580, and consists largely in a record of wonderful cures, wrought under the most daring and fantastic methods. Amongst many curious recipes is one for the making of a philosopher's stone, water distilled from which is guaranteed to cure all diseases "that happen unto man or woman, or any other animal terrestrial."

By far the most advanced work on surgery to which the Englishman had access, was that written by Jacques Guillemeau, and translated by A. M., oddly enough, from the Dutch, and printed at Dort in the year 1597. The dedication is to Queen Elizabeth, and the work is most comprehensive. Not the least interesting features are the elaborate engravings of the surgical instruments then most in use, with their names in Dutch and French. Guillemeau was one of the most enlightened surgeons of the age; a disciple of Ambroise Paré, he spared no pains to make himself expert by practice, and for many years exercised his art at the Hotel Dieu, where he gained a ripe experience. It is doubtful whether this magnificent folio can have circulated largely among the surgeon class.

A small treatise of Du Chesne must not be omitted among the foreign authorities on the surgeon's shelf. Du Chesne is credited with having held that the imaginary burn of gunshot wounds is the most important part of the accident. However that may be, his treatise on gunshot wounds was in high repute in England under the barbarous appellation of a "Sclopotarie."* It was translated in 1590 by the indefatigable John Hester, and again some years after. His spageric remedies include directions for distilling oils of mercury, of vitriol, of salt, of talc, of sulphur, and of antimony, and directions for preparing a much-esteemed water from the sperm of frogs.

Finally, the work on surgery of Jean de Vigo, whose strange medical theories have been already noticed, enjoyed a wide popularity, to judge from the numerous editions issued of the translation by Bartholomew Traheron in 1543. It treats of anatomy, wounds, ulcers, fractures, &c., in a simple and popular manner, but displays no very high standard of knowledge.

* Cf. Dom Hyacinth :—
Or might one style a pistol—popping-piece ?
Armatus breviori sclopulo ?—"The Ring and the Book."

